# Review of Techniques for Protecting Side Branch from Occlusion during Provisional Stenting in Coronary Bifurcation Lesions

**DOI:** 10.31083/j.rcm2411323

**Published:** 2023-11-23

**Authors:** Dongdong Li, Huimiao Dai, Chuncheng Gao, Hao Liu, Aili Yang, Wangang Guo

**Affiliations:** ^1^Department of Cardiology, Tangdu Hospital, Air Force Medical University, 710038 Xi'an, Shaanxi, China; ^2^Department of Endocrinology, Tangdu Hospital, Air Force Medical University, 710038 Xi'an, Shaanxi, China

**Keywords:** coronary bifurcation lesion, crossover stenting, MACE, provisional stenting, side branch occlusion

## Abstract

Coronary bifurcation lesions remain one of the most challenging lesions for cardiology interventionists. The provisional stenting strategy has been regarded as the first option for most of these lesions. However, the main complication of this technique is side branch (SB) occlusion, which could lead to a peri-procedural myocardial infarction or even death. Various studies have focused on addressing this issue, but there are no definitive guidelines in the literature to treat these lesions. There isn’t enough clinical evidence from randomized controlled trial or two-arm cohort studies to illustrate which techniques provide the best outcomes. In this review, we summarize the mechanisms, independent predictors and predictive models of SB occlusion, and review seventeen techniques involving SB protection and occlusion rescue. Every technique was evaluated according to related bench tests, clinical studies and our own clinical experiences. The aim of this review is to provide interventionists with new insights for the treatment of coronary bifurcation lesions.

## 1. Introduction

Side branch (SB) occlusion caused by carina or plaque shift is the main complication during the treatment of bifurcation lesions using the provisional stenting (PS) strategy [1]. Earlier studies have revealed that SB compromise during PS could be as high as 8.4–16% [2, 3]. Though rescue procedures could be performed to restore SB blood flow, prolonged SB compromise might result in peri-procedural myocardial infarction (MI) or even death, especially when the SB supplies a large and/or important territory of the myocardium. In addition, rescue maneuvers fail to restore blood flow on 31% of cases according to the COBIS II study [3]. Multiple strategies or techniques have been developed to solve this problem, including the jailed wire technique (JWT) [4], jailed balloon technique (JBT) [5, 6], modified jailed balloon technique (MJBT) [7], jailed semi-balloon technique (JSBT) [8], jailed corsair technique (JCT) [9], balloon-stent kissing technique (BSKT) [10], modified balloon-stent kissing technique (MBSKT) [11], double kissing inflation outside the stent technique (DKO) [12] and jail escape technique (JET) [13]. Other techniques, such as the rescue balloon jailed technique (RBJT) [14], rescue inverted crush technique (RICT) [15], repetitive proximal optimization technique sequences (rePOT) [16], double balloons kissing (DBK) followed by POT [17, 18] and proximal optimization with kissing balloon inflation technique (POKI) [19], provide effective alternatives for SB flow rescue or restoration. Khan *et al*. [20] reviewed seven techniques for SB protection in 2020, and then, no updated review was released. In this study, we made a systematic review aiming at clarifying all the techniques concerning SB protection, so as to provide choices for interventional cardiologists to deal with bifurcation lesions.

## 2. Mechanisms, Predictors and Risk Models of Side Branch Occlusion

### 2.1 Mechanisms of Side Branch Occlusion

**Fig. 1.**
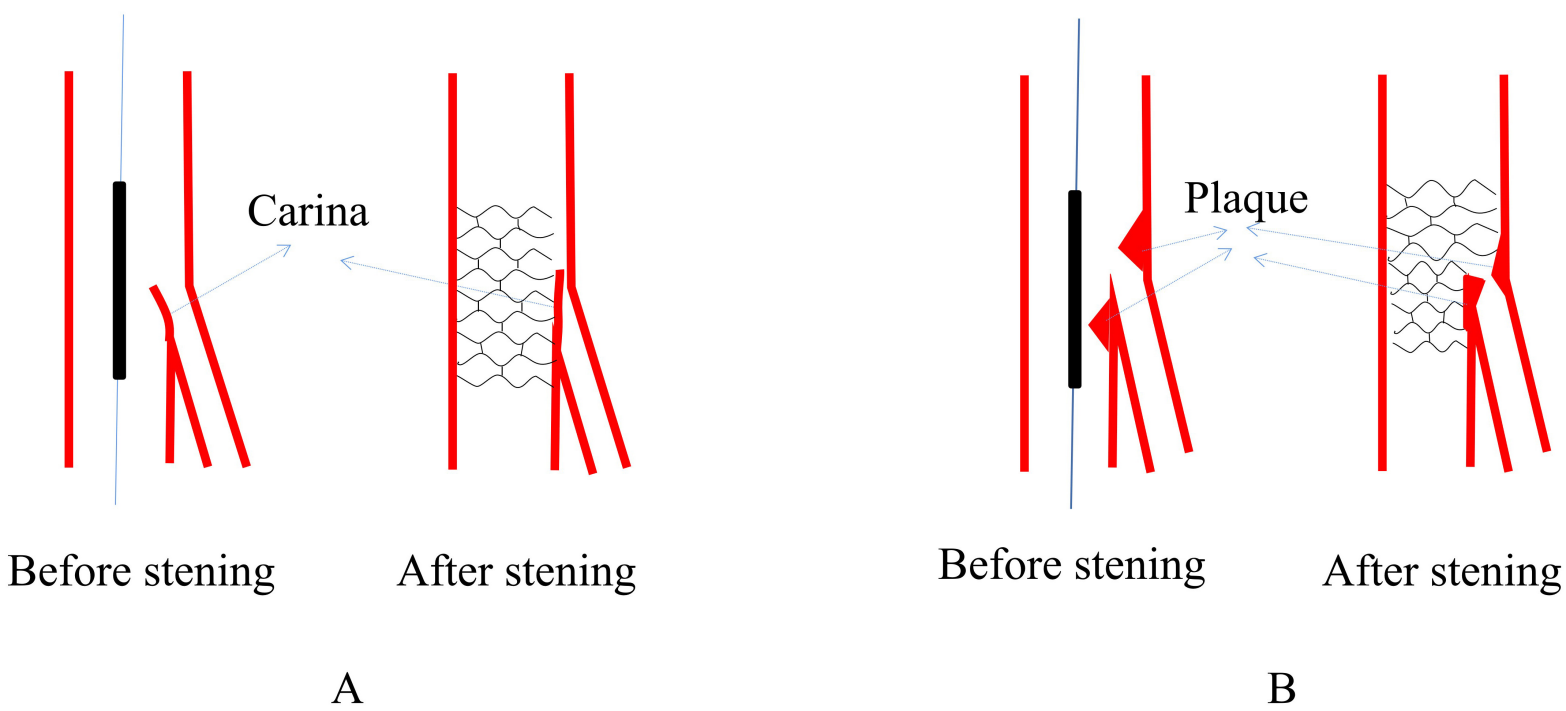
**Schematic presentation of two dominant mechanisms of SB occlusion**. (A) Carina shift happened on a bifurcation with long carina. (B) Plaque shift happened on a bifurcation with high plaque burden around SB ostium. SB, side branch.

The mechanisms of SB ostium stenosis and occlusion after main vessel (MV) stenting are carina shift, plaque shift, ostial dissection, thrombus formation, and spasm [21]. Carina and plaque shifts are the two dominant mechanisms [22]. Carina shift is mainly induced by stent overexpansion or the selection of an oversized stent relative to the distal MV, which could push the carina to cover the SB ostium (Fig. 1A). The carina mismatch model proposed by Vassilev *et al*. [23] nicely describes the procedure. The carina is like a door, and the SB ostium represents the door frame. When the door is wider than the frame, the frame is more likely to be closed. This explains why a bifurcation with sharp angle and small SB is more easily compromised in clinical practice. SB ostium stenosis or occlusion due to plaque shift usually occurs in the presence of large plaque burden around the SB ostium. The plaque on the bilateral sides of the SB could be compressed into the SB by the dilated stent similar to the snowplow phenomenon (Fig. 1B). Xu *et al*. [24] found that the carina shift was responsible for 85% cases of SB occlusions. However, a recent study using pressure wire techniques revealed that the carina shift did not result in a significant reduction in fractional flow reserve (FFR), but mainly lead to anatomical SB stenosis and not functional one. On the contrary, SB stenosis caused by plaque shift was always functionally significant [25]. This phenomenon might account for the acute thrombosis formation when the plaque was ruptured, and highlighted the importance of those risk factors responsible for plaque shift resulting in SB occlusion during MV stenting.

### 2.2 Predictors of Side Branch Occlusion

True bifurcation lesions (Medina 1,1,1; 1,0,1; 0,1,1) involve both MV and SB and are regarded as relatively high-risk lesions [26]. However, the medina classification only describes the site of the lesion and the severity of stenosis, which has little association with the carina shift. Dou *et al*. [27] revealed that the incidence of SB occlusion between true and non-true bifurcation lesions was not significantly different. More detailed factors and predictors of SB occlusion during MV have been reported (Table 1, Ref. [3, 26, 28, 29, 30, 31, 32, 33, 34, 35, 36]). These include: (1) Bifurcation angle. A larger bifurcation angle is representative of a longer carina. Carina displacement after MV stenting could completely cover the SB ostium if its diameter is relatively small. Dou *et al*. [28] revealed that a bifurcation angle >52° was an independent risk factor. The incidence of SB occlusion in lesions with an angle >52° could reach as high as 10.5%, and increased when the angle was increased. Medina *et al*. [29] found that intravascular ultrasound (IVUS) could detect the “eyebrow sign” another independent predictor, which described a long carina seen in the longitudinal view of IVUS. (2) Plaque burden around the SB ostium. A SB originating from a stenosed segment was more likely to be compromised when performing MV pre-dilation or stenting. (3) SB ratio and MV plaque sickness under IVUS observation. Sakamoto *et al*. [30] used IVUS and found two additional predictors. One was the SB diameter ratio defined as the SB total diameter/SB luminal diameter. The sensitivity of using a ratio ≥1.5 to predict SB occlusion was 85%, while the specificity was 66% [30]. A SB ostial stenosis could be increased following a carina or plaque shift. Therefore, SB pre-dilation was thought to be indicated under these circumstances. The other IVUS-detected predictor was MV plaque thickness ≥0.9 mm on the bilateral side of the SB. The sensitivity to predict SB occlusion was 71%, while the specificity reached 89%. Large MV plaque burden was more apt to cause the snowplow effect responsible for plaque shift. (4) Maximal lipid arc. Cao *et al*. [26] used optical coherence tomography (OCT) and found that the maximal lipid arc was another predictor. This pattern of plaque was more likely to rupture and result in SB occlusion [26, 31].

**Table 1.** **Studies concerning independent predictors of side branch occlusion**.

No.	Study	Design	Sample size (+/–)	Detecting method	SB involved	Outcome definition	Independent predictors
1.	Zhang, 2015 [28]	Retrospective/cohort	1200 (large angle 600/small angle 600)	angiography	Significant SB based on the operators’ discretion	SB occlusion was defined as absence of flow in the SB or any TIMI flow grade decrease in SB after MV stenting	Bifurcation angle
2.	Medina, 2009 [29]	Retrospective/case control	71 (7/64)	IVUS	LCX	-	Carina having a spiky appearance on IVUS (eyebrow sign).
3.	Sakamoto, 2016 [30]	Retrospective/case control	272 (52/220)	IVUS	SB with diameter ≥1.5 mm measured by IVUS	SB occlusion was defined as a TIMI of ≤2 grade flow	The thickness of MV plaque on the bilateral sides of SB at the junction site; the SB diameter ratio.
4.	Kini, 2017 [31]	Prospective/case control	30 (10/20)	OCT	Significant SB based on the operators’ discretion	Significant SB ostium stenosis defined as residual stenosis of >50%	maximal lipid arc; the presence of lipid plaque contra-lateral to SB ostium.
5.	Cao, 2019 [26]	Retrospective/case control	207 (26/181)	OCT	SB diameter ≥1.5 mm by angiographic visual estimation	SB occlusion was defined as TIMI flow grade 0/1	OCT-detected layered pattern; true bifurcation lesion; wider angiographic bifurcation angle.
6.	Dou, 2016 [36]	Retrospective/case control	1601 (118/1453)	QCA	Significant SB based on the operators’ discretion	-	plaque distribution; MV TIMI flow grade before stenting; pre-procedural diameter stenosis of bifurcation core; bifurcation angle; diameter ratio between MV/SB; diameter stenosis of the SB before MV stenting.
7.	Lee, 2019 [32]	Retrospective/case control	260 (42/218)	CTA	SB diameter ≥2.0 mm	SB occlusion was defined as development of SB flow with TIMI flow ≤1	SB plaque; calcified plaque in the MV; low attenuation plaque in the main proximal segment or SB; a ratio of MV to SB ostium area >4.3.
8.	Hahn, 2013 [3]	Retrospective/case control	2227 (187/2040)	QCA	SB with diameter ≥2.3 mm confirmed by QCA	SB occlusion was defined as a TIMI of <3 grade flow	Pre-procedural percent diameter stenosis of the SB ≥50%; the proximal MV ≥50%; SB lesion length; acute coronary syndrome.
9.	Lezo, 2012 [33]	Prospective/case control	110 (51/59)	IVUS	SB reference diameter was >2.25 mm; bifurcation coronary lesions that did not involve the SB ostium (Medina 1,0,0; 1,1,0; 0,1,0).	Ostial SB damage defined as an increase of the percentage of ostial stenosis by QCA ≥30%.	IVUS identified a carina with a spiky morphology (eyebrow sign); Narrower angiographic angles.
10.	Vassilev, 2008 [34]	Retrospective	57	QCA	SB with diameters greater than 2 mm.	-	Bifurcation angle
11.	Furukawa, 2005 [35]	Retrospective/cohort	81 (group 1: 20/group 2: 61)	IVUS	SB with an estimated reference luminal diameter of 1 mm or greater were considered.	SB occlusion was defined as a TIMI flow of ≤2	Ostial plaque distribution

CTA, computed tomography angiography; SB, side branch; MV, main vessel; OCT, optical coherence tomography; IVUS, intravascular ultrasound; TIMI, thrombolysis in myocardial infarction; QCA, Quantitative coronary angiographic; LCX, left circumflex.

### 2.3 Risk Models of Side Branch Occlusion

SB occlusion is the result of multiple factors. Dou *et al*. [36, 37] used the RESOLVE score system to stratify the risk of SB occlusion. A score of 0–43 was assigned to every bifurcation lesion after an evaluation of six factors (No. 6 in Table 1). A larger score indicated a higher risk. Other studies validated this system in both non-left main and left main bifurcations and documented its accuracy [38]. The six factors were assessed in at baseline and after the lesion was treated. Dou *et al*. [39] then simplified this process by just assessing all the lesions at baseline. The area under the receiver operator characteristic curve (AUC) was similar between the two systems (0.735* vs. *0.756, *p *= 0.191), so the simplified system had the same efficiency. They concluded that a score of 14–43 had a higher SB occlusion risk than a score less than 14 (17.31% *vs.* 4.74%, *p <* 0.01). In 2019, Opolski *et al*. [40] used computed tomography angiography (CTA) to predict occlusion risk. They applied the RESOLVE score system to CTA and achieved a comparable AUC (0.709 *vs.* 0.752, *p *= 0.531). Lee *et al*. [32] established another model with CTA involving four factors (No. 7 in Table 1), and found that this model performed better than the RESOLVE score system (0.746 *vs.* 0.627, *p* < 0.05).

## 3. Side Branch Protection Techniques

### 3.1 Jailed Wire Technique

JWT is the most widely used technique to place a guide wire in the SB during main vessel (MV) stenting and performing POT. The wire occupies the ostial space to prevent SB closure due to a carina or plaque shift. Once SB flow was compromised, the wire should act as a marker and angle modifier to facilitate SB rewiring for subsequent SB dilating, balloon kissing or even SB stenting. If the SB could not be crossed, a low-profile balloon could be advanced underneath the struts to the SB ostium to restore flow [41]. Compared with provisional stenting without a jailed wire, JWT reduced the SB occlusion rate [42]. However, results from COBIS II showed that the jailed wire did not reduce the rate of SB compromise except for providing a path to re-cross the SB using another guiding wire [3]. Rewiring the SB from the strut cell prolonged the operation time and increased the risk of SB dissection. Wire entrapment often occurs in severely calcified lesions and when stents were deployed under higher pressures. Retraction of the entrapped wire could lead to wire fracture, stent deformation or vessel injury [43, 44, 45]. Therefore, a jailed wire is more suitable for SB location during the treatment of SB with a low-risk of occlusion, characterized by a larger lumen diameter, low plaque burden, short carina length, and small bifurcation angle.

For those SB lesions in which placing a guidewire is difficult, such as extreme tortuosity, extreme angulation of the SB, and severe stenosis at the bifurcation, a steerable micro-catheter might be helpful. The operator could modify the catheter tip angle manually. Kassimis *et al*. [46] successfully advanced wires into complex SBs in two cases with steerable Venture and Swift Ninja micro-catheters. Cui *et al*. [47] used Crusade double-lumen microcatheter to perform reverse wire technique and successfully wired markedly angulated SBs. They applied this technique in 7 cases and successfully introduced the wire into the target vessel without any complications or major adverse cardiac events (MACEs) [47].

### 3.2 Jailed Balloon Technique by Burzotta et al.

**Fig. 2.**
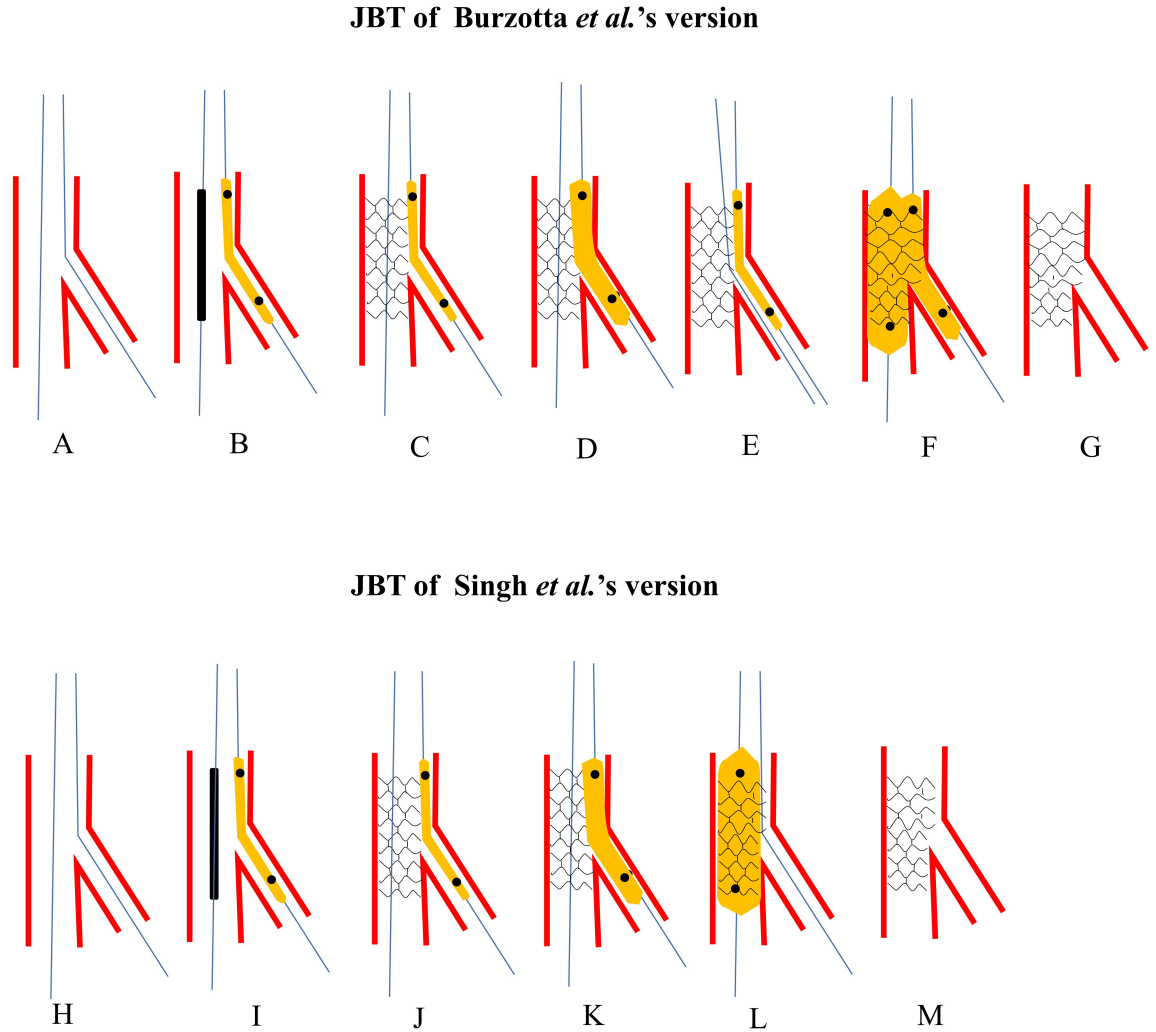
**Schematic presentation of JBT**. The upper panel: Burzotta *et al*.’s [5] version. (A) Both MV and SB were wired. (B) A small non-compliant balloon was jailed and the proximal marker was at the level of stent’s proximal edge. (C) The stent was deployed at nominal pressure. (D) Once SB was compromised and rewiring SB was failed, the jailed balloon would be inflated. (E) Rewiring SB with the jailed balloon as a marker and angle modifier. (F) DBK was performed. (G) Final stent morphology. The lower panel: Singh *et al*.’s [6] version. (H) Both MV and SB were wired. (I) A small non-compliant balloon was jailed and the proximal marker is at the level of stent’s proximal edge. (J) The stent is deployed at nominal pressure. (K) Once SB was compromised and rewiring SB was failed, the jailed balloon would be inflated. (L) Post-dilating the whole stent for better apposition. (M) Final stent morphology. JBT, jailed balloon technique; MV, main vessel; SB, side branch; DBK, double balloons kissing.

Burzotta *et al*. [5] first proposed the JBT in 2010, in which a non-inflated balloon was placed in the SB before MV stenting. If SB flow was less than TIMI 3, the jailed balloon acted as a marker to facilitate wire re-crossing SB. Once re-crossing failed, the jailed balloon could be inflated to quickly reopen the SB ostium (Fig. 2A–G, Ref. [5, 6]). A bench test was conducted and verified that balloon inflation could lead to stent malapposition, and a prompt post-dilation was mandated to reappose the stent strut. They used JBT in 20 patients with true bifurcation lesions, in whom three SB occlusions occurred after MV stenting. Two were rescued after SB rewiring and subsequent dilating, and one was rescued by inflating the jailed balloon. No balloon entrapment or dissection was observed.

Jailed balloon technique offered larger spatial occupation in SB ostium to prevent carina or plaque shift. It also provided better visible markers and efficient angle modifier for SB rewiring and made possible faster SB flow restoration by inflating the jailed balloon.

JBT also has certain risks. A major risk is stent malapposition in the proximal segment. Burzotta *et al*. [5] found that in a bench test, a non-inflated jailed balloon did not induce major malapposition. It only occurred after the balloon was inflated. The malapposition or distortion in this study was identified by direct vision, but not through OCT or IVUS. According to the latest OCT consensus, only malapposition ≥300 μm was clinically relative [48]. POT has been recommended as a final routine default in most of the provisional stentings, which could eliminate malapposition and stent deformation to an acceptable degree. Malapposition induced by JBT could be treated by POT.

The second risk of JBT is balloon entrapment or rupture. Numasawa *et al*. [49] reported a case of balloon entrapment in a calcified lesion. A high-quality balloon should be chosen as the jailed balloon, and the balloon’ length should be long enough to exceed the proximal stent edge. The balloon should be removed gently. Once balloon entrapment occurs, the balloon should be inflated and then removed gently.

In Burzotta *et al*.’s [5] protocol, JBT was finalized with a DBK. However, the latest studies have revealed that DBK led to elliptical stent deformation [50] and resulted in no clinical benefits [51]. Thus, DBK should be followed by POT, or replaced by rePOT.

### 3.3 Jailed Balloon Technique Proposed by Singh et al.

In the JBT by Singh *et al*. [6], the jailed balloon was routinely inflated at a low pressure before removal. The stent balloon was inflated with moderate or high pressure as the final step to optimize stent apposition and correct distortion by the jailed balloon (Fig. 2H–M). This technique was applied to 102 bifurcation lesions, most of which were classified as Medina 1,1,1. SB flow compromise only occurred in one case. No balloon entrapment, rupture or SB dissection was reported [6].

As opposed to Burzotta *et al*. [5], in Singh *et al*.’s [6] JBT, SB rewiring was not routinely performed, which simplified the procedure and saved time. According to the European Bifurcation Club (EBC) consensus, SB rewiring and DBK were only performed when SB flow was compromised [17]. The stent struts that cover over the SB ostium should not be cleared routinely [52].

Zhang *et al*. [53] conducted a randomized controlled trial to compare the efficacy of JWT and JBT. In that trial, 284 patients with a high risk of SB occlusion were randomly assigned to a JWT and JBT groups. In the JBT group, the technique of Singh *et al*. [6] was used, but the procedures were performed with a standard POT. The results favored the JBT group which had a significantly lower rate of SB occlusion (9.1% *vs.* 19.9%, *p *= 0.02) and a similar incidence of cardiac death, myocardial infarction (MI), target lesion revascularization (TLR) and MACE rates in one-year follow-up. The studies involving JBT are summarized in Table 2 (Ref. [5, 6, 53, 54]).

**Table 2.** **Studies involving jailed balloon technique**.

No.	Study	Design	Sample size (patients/lesions)	Lesion characters	SB occlusion (%)	MV dissection	SB dissection	Entrapment	Periprocedural MACE	Follow-up
1	Burzotta, 2010 [5]	Prospective/single arm	17/20	Medina 1,1,1 (85%);	3 (15%)	-	-	0	-	-
				Medina 0,1,1 (5%);						
				Medina 1,0,1 (10%)						
2	Singh, 2012 [6]	Retrospective/single arm	100/102	Medina 0,0,1 (0%);	1 (1%)	4 (4%)	0	0	MI: 1 (1%)	-
				Medina 0,1,0 (2%);						
				Medina 0,1,1 (2%);						
				Medina 1,0,0 (0%);						
				Medina 1,0,1 (1%);						
				Medina 1,1,0 (4%);						
				Medina 1,1,1 (93%)						
3	Depta, 2013 [54]	Retrospective/double arms (*vs.* Non-JBT)	95/98	Medina 0,0,1 (0%);	11 (11%)	-	-	-	MI: 1 (1%)	2.7 ± 2.1 years:
				Medina 0,1,0 (2%);						Death: 2 (2%);
				Medina 0,1,1 (2%);						MI: 1 (1%);
				Medina 1,0,0 (0%);						TLR: 2 (2%);
				Medina 1,0,1 (1%);						TVR: 4 (4%)
				Medina 1,1,0 (4%);						
				Medina 1,1,1 (91%)						
4	Zhang, 2022 [53]	RCT (*vs.* JWT)	143/143	Medina 0,0,1 (0%);	13 (9.1%)	-	-	-	MI: 10 (7.0%)	1 year:
				Medina 0,1,0 (1.4%);						MACE: 12 (8.4%);
				Medina 0,1,1 (16.1%);						Death: 1 (0.7%);
				Medina 1,0,0 (2.1%);						MI: 9 (6.3%);
				Medina 1,0,1 (10.5%);						TVR: 3 (2.1%);
				Medina 1,1,0 (7.7%);						
				Medina 1,1,1 (62.2%)						TLR: 2 (1.4%)

SB, side branch; MV, main vessel; JBT, jailed balloon technique; JWT, jailed wire technique; RCT, randomized controlled trails; MI, myocardial infarction; TLR, target lesion revascularization; TVR, target vessel revascularization; MACE, major adverse cardiac events.

### 3.4 Modified Jailed Balloon Technique

**Fig. 3.**
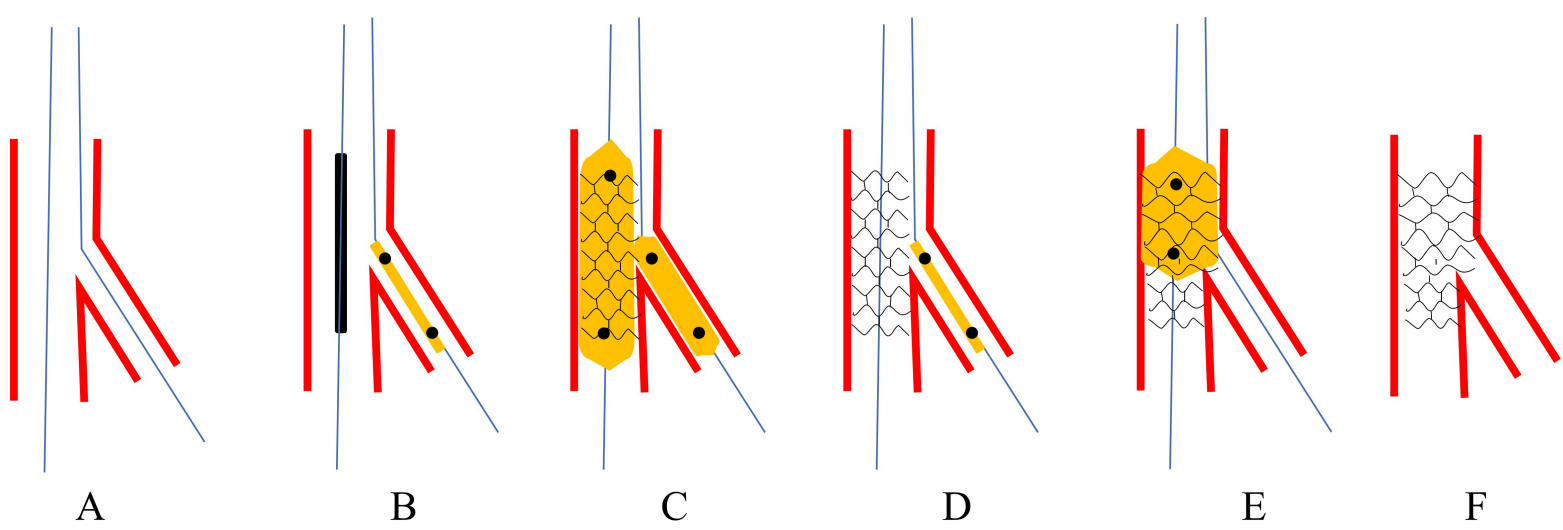
**Schematic presentation of MJBT**. (A) Both MV and SB were wired. (B) A balloon was jailed in SB and the proximal edge just attached to the strut. (C) The stent balloon and jailed balloon were inflated simultaneously. (D) Retrieving the two balloons. (E) Performing POT. (F) Final stent morphology. MJBT, modified jailed balloon technique; MV, main vessel; SB, side branch; POT, proximal optimization technique.

Saito *et al*. [7] proposed a MJBT in 2017. The key points were: a short balloon was totally introduced into SB; the balloon size was chosen as half of the MV stent diameter but no larger than the SB diameter; and the stent balloon and jailed balloon were inflated simultaneously at the same pressure during deploying the MV stent (Fig. 3).

This technique has some advantages. A short but thick balloon was chosen to be jailed in the SB for maximizing spatial occupation. The balloon was inflated simultaneously with the stent balloon to prevent carina or plaque shift. The balloon was totally advanced into the SB, and only the shaft was compressed under the stent strut, thus, less stent deformation was induced irrespective of the size of the jailed balloon and whether it was inflated.

Saito *et al*. [7] in a bench test showed that MJBT induced less stent deformation in the proximal segment compared with JBT (appraised by eccentricity index: 1.06 ± 0.02 *vs.* 1.11 ± 0.04, *p *= 0.009). A clinical study of 254 lesions in 233 patients revealed no critical narrowing of the SB or severe dissection. All balloons were removed smoothly, and no TLR occurred in the six-month follow-up. Saito *et al*. [7] reported the results of a long-term follow-up of 349 lesions in 328 patients who received MJBT in their institution since 2015. Four temporal SB loss, defined as less than thrombolysis in myocardial infarction (TIMI) 3 flow, occurred after MJBT caused by dissection or hematoma. In the median follow-up of 717 days, all-cause death was 7.0%, TLR was 5.8%, and MI was 0.6% [55]. Nomura *et al*. [56] showed that SB balloon pressure, or stent balloon nominal pressure did not affect procedural outcomes. However, SB dissection occurred in 14 of the 51 patients, SB flow less than TIMI 3 occurred in 3 lesions, and SB ostial stenosis increased in 5 patients [56].

These studies confirmed that it was feasible to retract the jailed balloon even though it was advanced deeply into the SB. However, the best-in-class balloons and stents were used in the cases by Saito *et al*. [7] Whether this could be reproduced using other balloons remains to be seen [57]. Another issue of concern was that SB dissection occurred both in the studies by Saito *et al*. [7] and Nomura *et al*. [56]. Balloon inflation was the main cause. MJBT preserved SB patency at the expense of increasing the risk of dissection. The studies involving JBT are summarized in Table 3 (Ref. [7, 55, 56]).

**Table 3.** **Studies involving modified jailed balloon technique**.

No.	Study	Design	Sample size (patients/lesions)	Lesion characters	SB occlusion (%)	MV dissection	SB dissection	Entrapment	Periprocedural MACE	Follow-up
1	Saito, 2018 [7]	-/single arm	233/254	Medina 1,1,1 (20.9%);	0	-	-	0	0	6 months:
				Medina 1,1,0 (16.5%);						TLR: 0
				Medina 1,0,1 (13%);						
				Medina 0,1,1 (26.8%);						
				Medina 1,0,0 (4.7%);						
				Medina 0,1,0 (17.3%);						
				Medina 0,0,1 (0.8%)						
2	Shishido, 2020 [55]	Retrospective/single arm	328/349	Medina 1,1,1 (49.0%);	4 (1.1%)	-	-	0	-	717 days:
				Medina 1,0,1 (14.9%);						TLR: 19 (5.8%);
				Medina 0,1,1 (36.1%)						All-cause death: 23 (7.0%);
										Cardiac death: 7 (2.1%);
										MACE: 41 (12.5%);
										ST: 0
3	Nomura, 2021 [56]	Retrospective/double arms	51/51	Medina 1,1,1 (62.7%);	16 (31.4%)	-	14 (27.5%)	0	0	-
				Medina 1,1,0 (3.9%);						
				Medina 1,0,1 (11.8%);						
				Medina 0,1,1 (19.6%);						
				Medina 0,0,1 (2.0%)						

SB, side branch; MV, main vessel; TLR, target lesion revascularization; MACE, major adverse cardiac events; ST, stent stenosis.

### 3.5 Jailed Semi-Inflated Balloon Technique

**Fig. 4.**
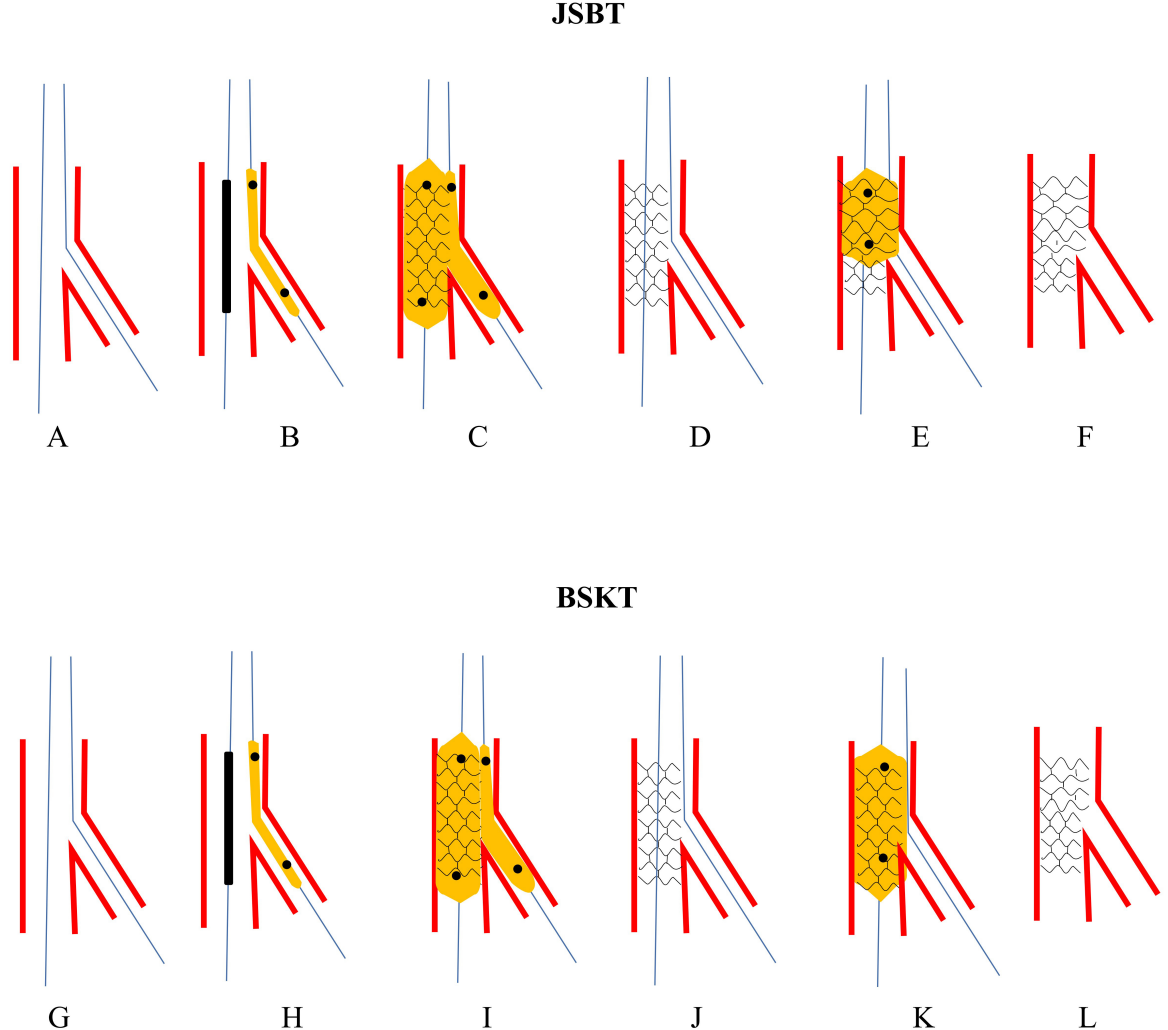
**The procedures of JSBT and BSKT**. Upper panel: Schematic presentation of JSBT. (A) Both MV and SB were wired. (B) A balloon was jailed and the distal edge just advanced into SB and inflated at a low pressure. (C) The stent balloon was dilated at nominal pressure. (D) Retrieving the two balloons. (E) Performing POT. (F) Final stent morphology. Lower panel: Schematic presentation of BSKT. (G) Both MV and SB were wired. (H) The distal edge of the jailed balloon was just advanced into SB and inflated at nominal pressure. (I) The stent balloon was dilated at nominal pressure. (J) Retrieving the two balloons. (K) Post-dilating the whole stent. (L) Final stent morphology. JSBT, jailed semi-inflation balloon technique; MV, main vessel; SB, side branch; POT, proximal optimization technique; BSKT, balloon stent kissing technique.

Çaylı *et al*. [8] introduced JSBT in 2015. In JSBT, a balloon was advanced into the SB, while a proximal balloon marker was placed at the same level at the stent balloon’s proximal edge. The jailed balloon was inflated at a low pressure, then the stent was dilated and squeezed the liquid inside jailed the balloon to the SB ostium. POT was finally implemented to optimize stent apposition (Fig. 4A–F).

Çaylı *et al*. [8] applied the JSBT to 148 lesions in 137 patients. TIMI 3 flow was established in all patients, although 4 patients developed an SB ostial dissection. All the balloons and wires were removed successfully. Ermiş *et al*. [58] applied JSBT to 64 patients with 82 lesions. SB ostial dissection was seen in 2 cases. No balloon or wire entrapment, and SB loss occurred, consistent with the study by Çaylı *et al*. [8]. No MACE was observed in the 1-month follow-up [58]. Su *et al*. [59] used the JSBT in 68 patients. SB dissection occurred in 8 cases. 4 cases underwent TVR, and 3 cases experienced an all-cause death in a median follow-up of 1.3 years [59].

JSBT achieved 100% TIMI 3 flow in these 3 studies. However, the incidence of dissection was increased. The MV and SB lumen sizes, plaque burden and operators’ skill level were variable. When a semi-compliant balloon was jailed, SB vessel dissection could not be totally avoided.

### 3.6 Balloon-Stent Kissing Technique

Jin *et al*. [10] introduced the BSKT in 2013. Similar to JSBT, the balloon jailed in SB was inflated before MV stenting, but the balloon inflation pressure was higher than in the JSBT. Therefore, post-dilation was conducted to optimize stent apposition (Fig. 4G–L). They applied the BSKT in 60 cases, 98% of which were true bifurcation lesions. All the SBs maintained a TIMI 3 flow after the procedures, and no balloon entrapment occurred. There were no SB dissections or peri-procedural MIs. Jin *et al*. [60] conducted a randomized controlled trial to compare the BSKT with JWT. In this study, no SB occlusion was observed after the procedures in the BSKT group, while there was an incidence of 15.6% in the JWT group. The perioperative MACEs were also significantly lower in the BSKT group compared to the JWT group. However, there was no significant difference in MACE in the mean 19-month follow-up period between the two groups.

Qu *et al*. [11] modified the BSKT (MBSKT) by finalizing the procedures with POT. Through an observation of a two-arm cohort study involving 40 patients who underwent MBSKT, Qu *et al*. [11] concluded that MBSKT was associated with a lower SB loss (3/40) compared with JWT (12/80). The incidence of MACE was similar to the JWT in the 12-month follow-up. No balloon entrapment was reported. Zhang *et al*. [61] tested the so-called “MJBT” in 60 patients. Actually, it was more likely to be MBSKT. TIMI flow less than 3 was found in 6.7% of the cases. All the balloons and wires were removed successfully and there was no MACE during a nine-month following-up..

### 3.7 Double Kissing Inflation Outside the Stent Technique 

Yang *et al*. [12] proposed the DKO technique in 2021. The procedure was the same as the MBSKT. They performed DKO on 117 patients. Procedural success was achieved in all patients. The studies involving JSIT, BSKT, MBSKT and DKO are summarized in Table 4 (Ref. [8, 10, 11, 12, 58, 59, 60, 61]).

**Table 4.** **Studies involving JSIT, BSKT, MBSKT and DKO**.

No.	Study	Design	Sample size (patients/lesions)	Lesion characters	SB occlusion (%)	MV dissection	SB dissection	Entrapment	Periprocedural MACE	Follow-up
1	Çaylı, 2015 [8]	-/single arm	138/147	Medina 1,1,1 (62.8%);	0	5 (3.4%)	6 (4.1%)	0	0	1 month:
				Medina 1,1,0 (18.2%);						MACE: 0
				Medina 1,0,1 (8.8%);						
				Medina 0,1,1 (2.0%);						
				Medina 1,0,0 (2.7%);						
				Medina 0,1,0 (5.4%)						
2	Ermiş, 2018 [58]	Prospective/single arm	64/82	Medina 1,1,1 (25.6%);	0	-	2 (2.4%)	0	0	1 months:
				Medina 1,1,0 (18.3%);						MACE: 0
				Medina 1,0,1 (12.2%);						
				Medina 0,1,1 (14.6%);						
				Medina 0,1,0 (4.9%)						
				Medina 1,0,0 (2.4%)						
3	Su, 2019 [59]	Retrospective/single arm	68/68	Medina 1,1,1 (64.7%);	0	-	8 (11.8%)	0	Death: 1 (1.5%)	1.3 years:
				Medina 1,0,1 (8.8%);						TLF: 0;
				Medina 0,1,1 (11.8%);						TLR: 0;
				Medina 1,1,0 (14.7%)						TVR: 4 (5.9%);
										MI: 0;
										All-cause death: 3 (4.4%)
4	Jin, 2013 [10]	Retrospective/single arm	60/60	-	0	2	0	0	0	-
5	Jin, 2019 [60]	RCT (*vs.* JWT)	44/45	Medina 1,1,1 (60.0%);	0	0	1 (2.2%)	0	0	2 years:
				Medina 1,0,1 (20.0%);						MACE: 3 (6.8%);
				Medina 0,1,1 (20.0%)						Cardiac death: 1 (2.3%);
										MI: 2 (4.5%);
										TLR: 0;
										Angina pectoris: 6 (13.6%);
										Severe heart failure: 1 (2.3%).
6	Qu, 2019 [11]	Prospective/double arms (*vs.* JWT)	40/40	Medina 1,1,1 (77.5%);	3 (7.5%)	0	0	0	-	1 year:
				Medina 1,0,1 (12.5%);						Stable condition: 37 (92.5%);
				Medina 0,1,1 (10.0%)						Rehospitalization for unstable angina: 3 (7.5%);
										MACE: 0
7	Yang, 2021 [12]	-/single arm	117/117	Medina 1,1,1 (98.3%);	1 (0.9%)	0	1 (0.9%)	0	0	-
				Medina 1,0,1 (1.7%);						
8	Zhang, 2019 [61]	Retrospective/single arm	60/60	Medina 1,1,1 (71.7%);	4 (15%)	-	0	0	0	9 months:
				Medina 1,0,1 (11.7%);						MACE: 0
				Medina 0,1,1 (16.7%)						

JSIT, jailed semi-inflation technique; BSKT, balloon stent kissing technique; MBSKT, modified balloon stent kissing technique; DKO, Double kissing inflation outside the stent technique; SB, side branch; MV, main vessel; TLF, target lesion failure; TLR, target lesion revascularization; TVR, target vessel revascularization; MACE, major adverse cardiac events; MI, myocardial infarction; RCT, randomized controlled trail; JWT, jailed wile technique.

### 3.8 Jailed Corsair Technique

**Fig. 5.**
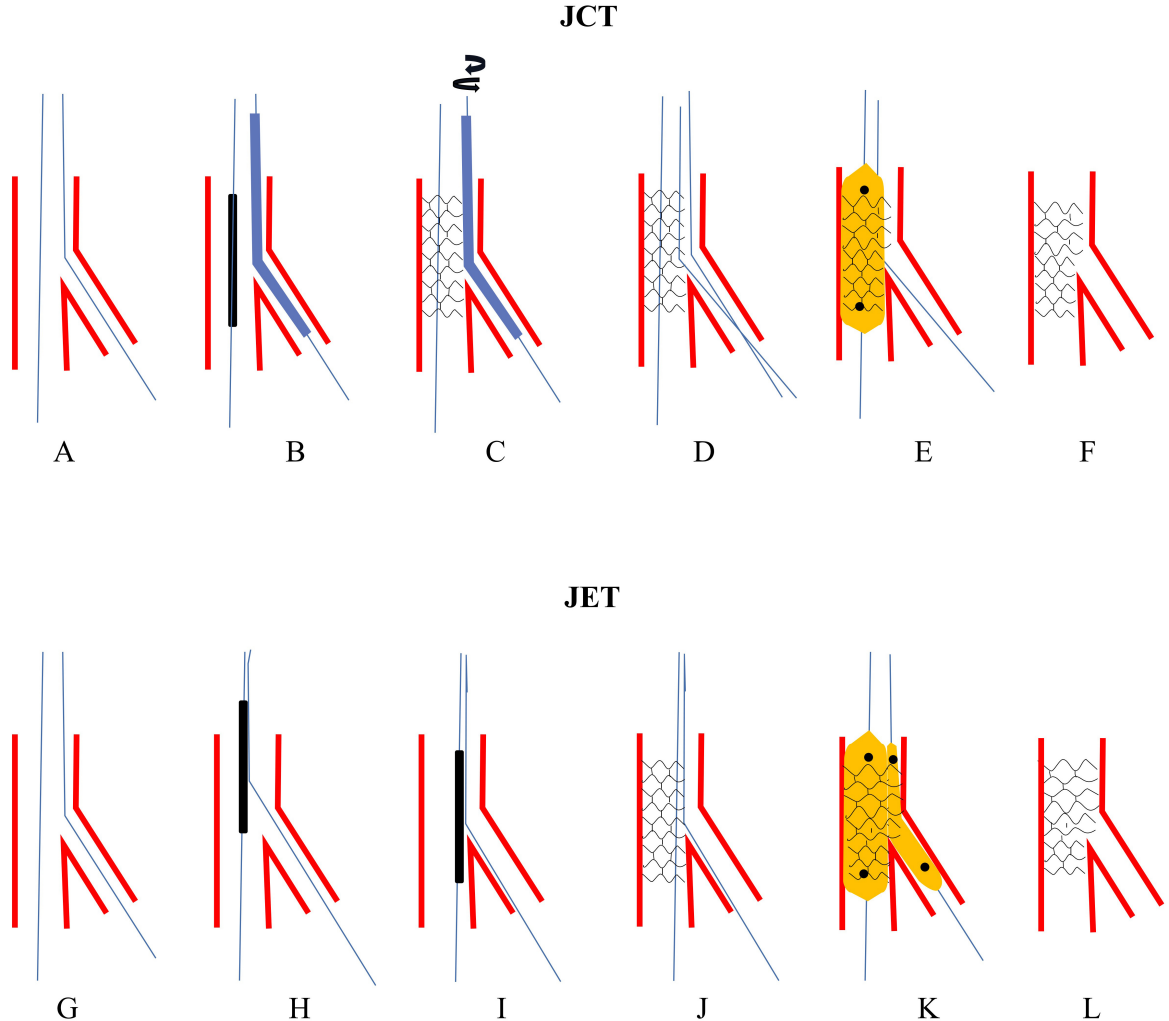
**The procedures of JCT and JET**. Upper panel: schematic presentation of JCT. (A) Both MV and SB were wired. (B) A Corsair microcatheter was jailed in SB. (C) The stent balloon was dilated at nominal pressure and the catheter was removed by rotating the shaft. (D) Rewiring SB with jailed wire acted as a marker. (E) Post-dilating the whole stent. (F) Final stent morphology. Lower panel: schematic presentation of JET. (G) Both MV and SB were wired. (H) Pass the proximal tip of the SB wire through the gap of stent strut and balloon from a cell in the middle strut. (I) Advance the stent through the two wires to the bifurcation. (J) Dilate the stent. (K) Perform DBK. (L) Final stent morphology. JCT, jailed corsair technique; MV, main vessel; SB, side branch; JET, jailed escape technique.

Numasawa *et al*. [9] reported one case in which the JCT was applied to protect a diagonal branch in a diffusely calcified stenosed LAD (Fig. 5A–F). In that case, a Corsair microcatheter was jailed in the SB before MV stenting. After the stent was deployed, the corsair was removed by rotating the shaft. Compared with JWT, jailed Corsair microcatheter occupied more space and was more easily removed. Compared with JBTs, the risk of ostium dissection was reduced.

### 3.9 Jailed Escape Technique

Xiao *et al*. [13] proposed JET in 2017 (Fig. 5G–L). They penetrated the tail of the SB wire into the undeployed stent underneath the strut before the stent was sent into the guiding catheter, so that re-crossing the SB wire would not be necessary. In this study, JET was performed successfully in 30 of the 32 cases. The two cases failed because of misalignment of the SB wire from the SB ostium. However, stent advancement over two wires could meet resistance, especially in severe, stenotic or calcified lesions. Procedure failure might occur because of wire fracture and/or stent dislodgement. Fischell *et al*. [62] noted that JET led to potential medico-legal issues for not following the instructions published for a particular stent delivery system.

### 3.10 Jailed Balloon-Proximal Optimization Technique

**Fig. 6.**
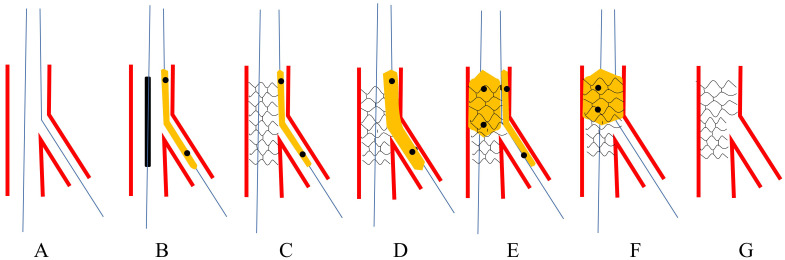
**Schematic presentation of JB-POT**. (A) Both MV and SB were wired. (B) A small non-compliant balloon was jailed and the proximal marker was at the level of stent’s proximal edge. (C) The stent was deployed at nominal pressure. (D) Once SB was compromised, the jailed balloon would be inflated. (E) POT was performed while jailed balloon was left in SB. (F) Second POT was performed at the take-off level after removing the jailed balloon. (G) Final stent morphology. JB-POT, jailed balloon-proximal optimization technique; MV, main vessel; SB, side branch.

When performing POT, there is also the risk of SB occlusion. If the POT balloon is positioned too distally, the vessel will be overstretched and carina shift will occur. POT could also further compress the MV plaque into the SB. EBC recommended keeping the jailed wire in the SB as a marker if re-cross is needed. Some interventionalists are used to re-crossing the SB before performing POT in case re-crossing fails after POT. However, re-crossing the SB is difficult and time-consuming, especially from a distal strut cell. Hence, we proposed jailed balloon-proximal optimization technique (JB-POT) to effectively address this problem (Fig. 6) [63]. In the JB-POT protocol, the jailed balloon is kept in the SB until the POT is concluded, which prevents carina and plaque shifts. If SB compromise still occurs, the jailed balloon could be inflated to restore SB blood flow. A rePOT away from SB take-off level should be performed as the final step to correct underlying stent malapposition. The advantages of JB-POT are the reduction in SB occlusion and the avoidance of the need for re-crossing the SB. The risks of it have been well studied in our bench test and clinical case series as well, like proximal stent malapposition, balloon entrapment, or dissection. In our bench test, we found that no major malapposition occurred even when the jailed balloon was inflated in the JB-POT maneuver by OCT measurement. This was due to the elastic character of the vessel wall and re-POT correction. In the 30 bifurcation lesions of the 28 case series, all SBs were well protected free of complications and rewiring SB was not required in most cases. With the conventional technique, repeated rewiring to protect all the SBs might lead to device entanglement and increase the risk of complications. JB-POT avoided rewiring step, reducing operation time and the consumption of contrast reagents, especially for lesions with multiple high-risk SBs.

## 4. Side Branch Rescue Techniques

### 4.1 Double Balloons Kissing Followed by POT

DBK had been a routine procedure to improve SB access and stent apposition. However, bench tests revealed that it caused proximal stent elliptical deformation, malapposition and vessel overstretch [16]. Clinical studies found that DBK had little clinical benefit and increased the incidence of TLR [52, 64, 65, 66, 67]. Based on these findings, the EBC recommended DBK only as a bailout method when SB required further intervention. A final POT must be added to correct MV deformation and apposition [17]. Dérimay *et al*. [50] reported that the final DBK could not completely correct the elliptical deformation and thus recommended the POT-side-rePOT protocol. However, more clinical data is required.

### 4.2 Repetitive POT Sequences

In view of the limitations of DBK, a new protocol including POT, SB dilation, and final POT was proposed, which showed better results in maintaining stent circular geometry and apposition [16]. Çetinkal *et al*. [68] showed a lower incidence of SB dissection and SB stenting compared with DBK. Bench testing found that SB dilation could lead to malapposition of the stent opposed to the SB ostium. Therefore, the final POT must be performed to re-oppose the stent. Kume *et al*. [69] found that the malapposition resulting from SB dilation was mainly caused by the long SB balloon which could bend the strut when inflated, thus a final POT would push the struts back to its original shape. These were also verified in the bench studies by Finet *et al*. [16] and Kume *et al*. [70]. Kume *et al*. [69] proposed an ultra-short balloon to dilate the SB located in the proximal end at the ostium, and named this strategy the “proximal balloon edge dilation” technique (PBEDT). PBEDT avoided bending the strut and improved the SB access, therefore apposition was acceptable and re-POT could be unnecessary. This technique requires further evaluation in multi-center clinical trials.

### 4.3 Proximal Optimization with the Kissing Balloon Inflation Technique

Vassilev *et al*. [19] proposed the POKI technique in 2022, which combined POT and DBK in one step. They put a POT balloon in the MV and SB balloon protruding into the MV, and then inflated the two balloons simultaneously.

POKI appeared to be a very promising technique, since it had all the advantages of POT and DBK, such as optimal apposition of proximal struts, facilitating SB rewiring from the distal cell, complete clearance of SB ostium struts and maximal SB ostium stent apposition. The disadvantages of DBK and rePOT could be avoided in this protocol, such as stent malapposition of the polygon zone and the increased incidence of obstruction of SB ostium by rePOT.

Vassilev *et al*. [19] applied POKI to 41 lesions, and all of them achieved procedural and angiographic success. The limitations of this study were that bench testing did not evaluate stent morphology and vessel model geometry, and that intravascular imaging was not performed in clinical trials.

### 4.4 Rescue Balloon Jailed Technique 

**Fig. 7.**
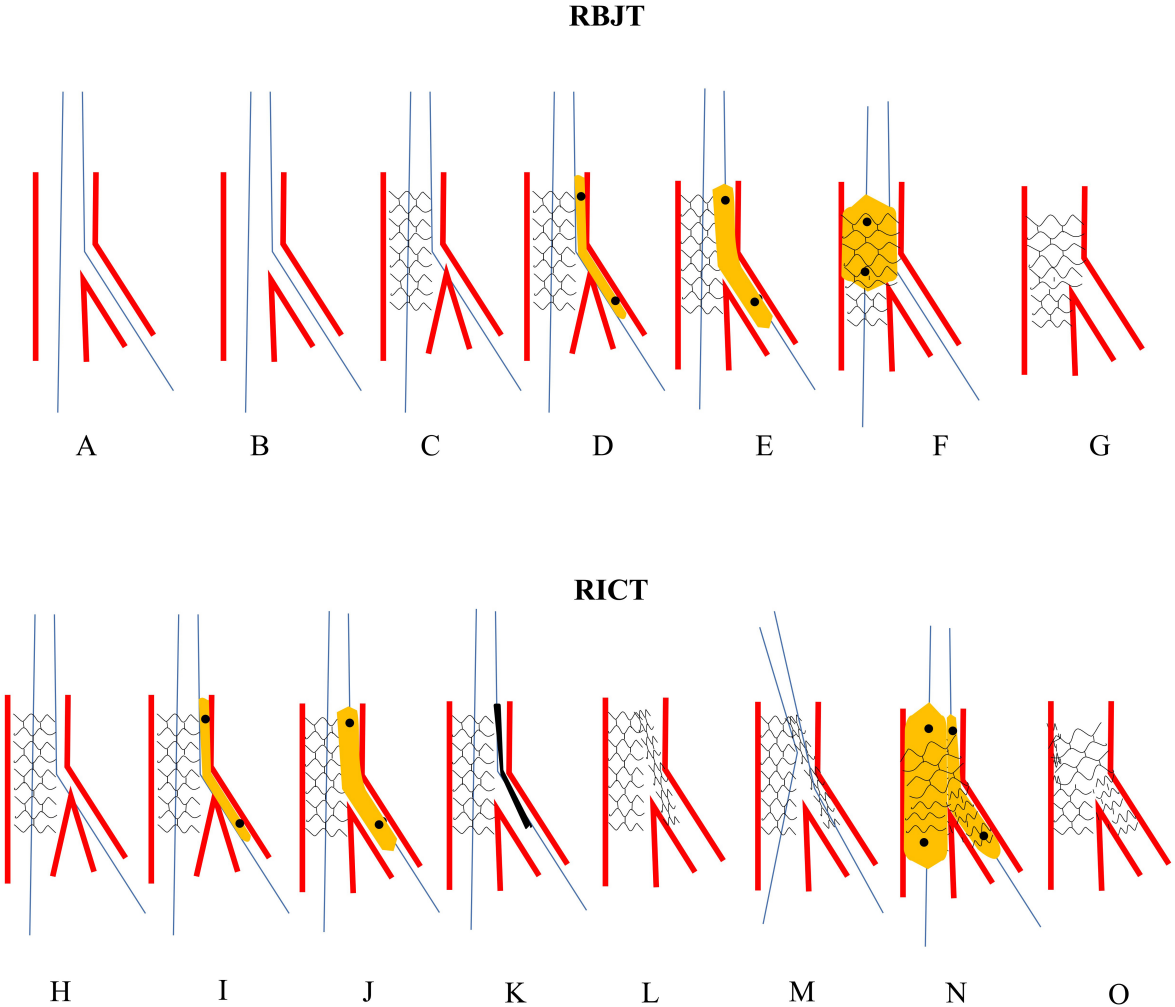
**The procedures of RBJT and RICT**. Upper panel: Schematic presentation of RBJT. (A) Both MV and SB were wired. (B) The stent was advanced to cross over the stent. (C) SB closed after MV stenting. (D) A low-profile and small balloon was forcefully advanced to the SB over the jailed wire. (E) The jailed balloon was gently inflated to regain access to SB. (F) POT was performed to correct the distortion of MV stent. (G) Final stent morphology. Lower panel: Schematic presentation of RICT. (H) SB closed after MV stenting. (I) A low-profile and small balloon was forcefully advanced to the SB over the jailed wire. (J) The balloon was inflated to crush MV stent to the opposite vessel wall. (K) A stent was introduced to SB. (L) The SB stent was deployed. (M) A wire was advanced to SB through 3-layer struts. (N) DBK. (O) Final stent morphology. RBJT, rescue balloon jailed technique; MV, main vessel; SB, side branch; POT, proximal optimization technique; RICT, rescue (inverted) crush technique; DBK, double balloons kissing.

During provisional stenting with JWT, rewiring SB tended to be difficult or even impossible when SB was totally closed or an ostial dissection occurred. Aminian *et al*. [14] introduced RBJT to a completely compromised SB. In RBJT, a low-profile and small balloon was forcefully advanced into the SB over the jailed wire. Then the balloon was gently inflated to regain access to the SB for SB recrossing and subsequent SB dilation or stenting. POT is mandated to correct the distortion of the MV stent (Fig. 7A–G) [42]. RBJT can be also be applied to retrieve a jailed wire which was entrapped underneath the stent struts. Sakamoto *et al*. [44] reported 28 patients who developed SB wire entrapment after MV stenting. Through RBJT, all the wires were removed, and 12-month MACEs were not observed in any of the cases [44].

### 4.5 Rescue (Inverted) Crush Technique

After rescuing the SB with RBJT, a severe dissection might develop, and SB stenting must be performed. Rewiring SB through the stent strut to perform a provisional T, TAP or culotte would be extremely difficult. In this situation, RICT tended to be appropriate and feasible. We can open an access underneath the MV stent by RBJT, through which the SB stent could be introduced into the SB. Then a rescue CRUSH or inverted CRUSH could be performed crushing the SB stent or MV stent by the NC balloon (Fig. 7H–O) [45, 71].

## 5. Discussion

In clinical practice, it is usually subjective for operators to evaluate whether a side branch is clinically significant. This involves several factors, including the patient’s symptoms, comorbidities, diameter and length of the side branch, plaque burden and localization, bifurcation angle, dominance size, location of ischemia, viability of the supplied myocardium, collateralizing of the vessel, left ventricular function, and the results of functional tests [72]. Among these factors, we think that the dominance of SB is the most important. The scaling law of V = ${\mathrm{KD}}^{2/3}$L tells us that the mass is positively correlated with the vessel diameter and length [73]. From the 14th EBC consensus, we know that SBs with length measured by computerized tomography angiography >73 mm were most likely to supply at least 10% of functional myocardial mass [74]. This allows us to evaluate the clinical significance of the vessel and then to choose the appropriate protection strategy.

From the detailed description of these techniques, we know that JBT has been the most effective technique to prevent SB compromise in provisional stenting. JBT was further modified by MJBT, JSIT, BSKT and MBSKT according to the balloon position, inflation pressure, and inflation timing. In general, the jailed SB balloon could be inflated or deflated depending on whether the SB blood flow was compromised by the traditional JBT or modified JBT. The jailed balloon must be inflated simultaneously with the MV stent deployment in BSKT, to decrease the possibility of SB compromise after MV stenting. But BSKT does increase unnecessary SB dilation and correspondent SB dissection. Repetitive POT must be performed to rectify the deformation induced by SB dilation or kissing. SB wire recrossing must be performed before post-dilating stents and POT, which increases the probability of device entanglement and procedure failure when there are multiple risk factors for SBs needing JBT protection. The JB-POT strategy partly solves this problem. Because the jailed balloon effectively prevents SB compromise induced by MV stenting, post-dilation and POT maneuvers, unnecessary SB rewiring and subsequent SB dilation and kissing are avoided. When JBT was required repeatedly in one procedure, JB-POT could help simplify the procedure process. Interventionists should be aware of these preventive strategies of SB loss and choose the appropriate technique in clinical practice.

When SB occlusion happened, rescue skills are critical to restore the SB blood flow. Micro-catheter, double lumen micro-catheter, chronic total occlusion guiding wires might help in recrossing. If rewiring failed, jailed balloon dilation could help wire recrossing SB ostium strut cells. Rescue crush (inverted) double stenting could be performed after dilation of the path to SB underneath the MV stent [75]. If SB rewiring got successful, SB-kissing or POT-side-rePOT could be applied according to the 15th EBC consensus. Provisional TAP, culotte or reverse culotte could be options of rescue double stenting when rewiring was obtained.

Aranzulla *et al*. [57] said: “True care is protecting who is at your side”. In determining how to achieve the best preservation of the SB, no strategy is perfect. The published data gives interventional cardiologists meaningful skills to obtain, and provides new sights for the treatment of coronary bifurcation lesions.

## 6. Conclusions 

This review summarizes the mechanism, independent predictors and risk models of SB stenosis/occlusion after MV stenting. Different types of SB protection and rescue techniques were described and discussed. There is still an absence of robust clinical data to determine which techniques are best. This review provides interventional cardiologists with alternative techniques to choose when dealing with the treatment of bifurcation lesions.

## Abbreviations

SB, side branch; MI, myocardial infarction; JWT, jailed wire technique; JBT, jailed balloon technique; MJBT, modified jailed balloon technique; JSBT, jailed semi-balloon technique; JCT, jailed corsair technique; BSKT, balloon-stent kissing technique; MBSKT, modified balloon-stent kissing technique; DKO, double kissing inflation outside the stent technique; JET, jail escape technique; RBJT, rescue balloon jailed technique; RICT, rescue inverted crush technique; re-POT, repetitive proximal optimization technique; DBK, double balloons kissing; POKI, proximal optimization with kissing balloon inflation technique; FFR, fractional flow reserve; IVUS, intravascular ultrasound; OCT, optical coherence tomography; ROC, receiver operator characteristic curve; AUC, area under ROC; CTA, computed tomography angiography; MV, main vessel; JB-POT, jailed balloon-proximal optimization technique; TLR, target lesion revascularization; MACE, major adverse cardiac events; TIMI, thrombolysis in myocardial infarction; PBED, proximal balloon edge dilation.
